# Treated and Untreated Primary Progressive Multiple Sclerosis: Walkthrough Immunological Changes of Monocytes and T Regulatory Cells

**DOI:** 10.3390/biomedicines12020464

**Published:** 2024-02-19

**Authors:** Nina Ipavec, Maja Rogić Vidaković, Anita Markotić, Sanda Pavelin, Maja Buljubašić Šoda, Joško Šoda, Krešimir Dolić, Nikolina Režić Mužinić

**Affiliations:** 1 Transfusion Medicine Division, University Hospital of Split, 21000 Split, Croatia; nina.ipavec@gmail.com; 2 Laboratory for Human and Experimental Neurophysiology, Department of Neuroscience, School of Medicine, University of Split, 21000 Split, Croatia; 3 Department of Medical Chemistry and Biochemistry, School of Medicine, University of Split, 21000 Split, Croatia; anita.markotic@mefst.hr; 4 Department of Neurology, University Hospital of Split, 21000 Split, Croatia; sanda.pavelin@mefst.hr; 5 Department of Pediatrics, University Hospital of Split, 21000 Split, Croatia; mbuljuba@kbsplit.hr; 6 Signal Processing, Analysis, Advanced Diagnostics Research and Education Laboratory (SPAADREL), Department for Marine Electrical Engineering and Information Technologies, Faculty of Maritime Studies, University of Split, 21000 Split, Croatia; jsoda@pfst.hr; 7 Department of Interventional and Diagnostic Radiology, University Hospital of Split, 21000 Split, Croatia; kdolic79@gmail.com; 8 Department of Radiology, School of Medicine, University of Split, 21000 Split, Croatia

**Keywords:** PPMS, regulatory T cells (Tregs), lymphocytes, Forkhead box protein P3, CTLA-4, monocyte subpopulations, ocrelizumab

## Abstract

The objective of this study was to investigate regulatory T cells (Tregs) and monocytes; specifically, the expression of CTLA-4 (CD152) and FOXP3^+^ in CD4^+^CD25^+^ Tregs and the expression of CD40+ and CD192+ monocyte subpopulations in subjects with primary progressive multiple sclerosis (PPMS). Immunological analysis was conducted on peripheral blood samples collected from the 28 PPMS subjects (15 treated with ocrelizumab and 13 untreated PPMS subjects) and 10 healthy control subjects (HCs). The blood samples were incubated with antihuman CD14, CD16, CD40, and CD192 antibodies for monocytes and antihuman CD4, CD25, FOXP3, and CTLA-4 antibodies for lymphocytes. The study results showed that in comparison to HCs both ocrelizumab treated (N = 15) and untreated (N = 13) PPMS subjects had significantly increased percentages of CTLA-4^+^ and FOXP3^+^ in CD4^+^CD25^+^ Tregs. Further, ocrelizumab treated PPMS subjects, compared to the untreated ones, had significantly decreased percentages of CD192+ and CD40+ nonclassical monocytes. Increased percentages of CTLA-4^+^ and FOXP3^+^ in CD4^+^CD25^+^ Tregs in both ocrelizumab treated and untreated PPMS subjects indicates the suppressive (inhibitory) role of Tregs in abnormal immune responses in PPMS subjects. Decreased percentages of CD40+ and CD192+ non-classical CD14^+^CD16^++^ monocytes for treated compared to untreated PPMS subjects suggest a possible role for ocrelizumab in dampening CNS inflammation.

## 1. Introduction

Multiple sclerosis (MS) is a disease characterized by multifocal demyelination leading to progressive neurodegeneration. It is caused by an autoimmune response directed against self-antigens within the central nervous system [1]. MS predominantly affects female subjects, with symptoms often starting to present in early adulthood. MS can be classified into two primary forms: the more common relapsing–remitting MS (RRMS), which is seen in about 85–90% of cases, and primary progressive MS (PPMS) [2]. RRMS exhibits cycles of neurological setbacks (relapses) followed by remission phases. However, eventually, most RRMS cases evolve into the consistently deteriorating condition known as secondary progressive MS (SPMS). On the other hand, the rarer form, PPMS, accounting for about 10–20% of MS diagnoses, presents as a persistent decline in neurological function from its onset, without periods of remission [3]. The fundamental pathological occurrence in MS is demyelination, accompanied by axonal degeneration and loss, leading to permanent functional impairments [4,5,6]. From an immunological perspective, MS arises due to aberrant immune system activation. Autoreactive CD4+ T cells bypass both negative selection and clonal elimination and penetrate the compromised blood–brain barrier to access the CNS and initiate the attraction of other inflammatory agents, including microglia, macrophages, and B cells. This cascade promotes antibody production and the release of proinflammatory cytokines, ultimately damaging the myelin sheath [7,8,9]. It is well established that autoreactive T cells play an important role in MS, as well as in other conditions such as psoriasis, diabetes mellitus type 1, oral lichen planus, myasthenia gravis, etc. [8,10,11,12,13]. Regulatory T cells (Tregs), a specific subset of T cells, are functionally changed in different autoimmune diseases [14,15,16,17,18,19,20], including MS [21,22,23,24,25,26]. Treg cells are distinguished as a CD4+ T cell subset that express the interleukin-2 receptor alpha chain CD25 and the pivotal transcription factor Forkhead box protein P3 (FOXP3). The presence of FOXP3 is vital for Treg cell development, functionality, and stability [27,28,29,30]. Also, Tregs markedly express the immune checkpoint receptor cytotoxic T-lymphocyte-associated antigen-4 (CTLA-4), also known as CD152. A deficiency in Treg-associated CTLA-4 can result in severe systemic autoimmune reactions [31,32]. Treg cells inhibit the activity of numerous cell types, including cytotoxic CD8+ T cells (Teffs) and antigen-presenting cells (APC). Tregs employ a variety of mechanisms to achieve inhibition of various cells (Teffs, APCs, etc.) ranging from direct cell-to-cell interactions to the release of suppressive cytokines [33].

Besides the role of autoreactive T cells in MS, monocytes and macrophages also contribute to proinflammatory and anti-inflammatory responses [34,35]. Monocytes are classified into three distinct subpopulations: classical (CD14^++^CD16^−^), intermediate (CD14^++^CD16^+^), and nonclassical (CD14^+^CD16^++^). Gjelstrup et al. [36] reported an increase in nonclassical monocytes, accompanied by a notable decrease in classical monocytes, as well as variations in the expression of CD40 and CD192, among MS subjects when compared to controls.

There are different treatment approaches for MS depending on the patient’s clinical symptoms. They may include treatment of relapse symptoms with steroid drugs and reducing the number of relapses with disease-modifying drugs (DMD) [37]. Ocrelizumab stands out as the sole approved DMD for PPMS. It reduces the progression of clinical impairment by around 25% and improves both clinical and magnetic resonance imaging (MRI) indicators of inflammation and degeneration in PPMS [38].

The present study aimed to investigate the expression of CTLA-4^+^ and FOXP3^+^ in CD4^+^CD25^+^ in Tregs, and CD40 and CD192 in the classical (CD14^++^CD16^−^), intermediate (CD14^++^CD16^+^), and nonclassical (CD14^+^CD16^++^) monocyte subsets in PPMS (ocrelizumab treated and untreated) subjects.

## 2. Materials and Methods

### 2.1. Participants

The study sample included twenty-eight PPMS subjects recruited from the University Hospital of Split, Croatia, who met the inclusion criteria and agreed to participate in the study. The healthy control group (HCs) comprised ten subjects. Fifteen out of twenty-eight PPMS subjects were treated with ocrelizumab (Ocrevus, Roche, Grenzach-Wyhlen, Germany) for ≥12 months. The ocrelizumab dosage of 600 mg was administered per month. Thirteen PPMS subjects refused the treatment (untreated PPMS). The mean age of the PPMS subjects was 54.57 ± 8.71, and for HCs the mean age was 37 ± 13.9. The mean age of the PPMS subjects was significantly higher (*p* < 0.003) compared to HCs. Most PPMS subjects were women (75%). The mean disease duration was 10.74 ± 7.57 years and the median Expanded Disability Status Scale (EDSS) score was 4.94. The duration of the MS disease was significantly longer in untreated PPMS subjects (*p* < 0.01) compared to ocrelizumab treated PPMS subjects. No significant differences were evident in EDSS scores between ocrelizumab treated and untreated PPMS subjects. Table 1 presents the basic demographic and disease related characteristics of PPMS subjects and HCs submitted to the immunological analysis.

### 2.2. Data Collection Procedures: Peripheral Blood (PB) Collection, Flow Cytometry, and Clinical Assessment (Neurological)

PB was collected, followed by a neurological examination on the same day. Collection of PB and neurological examination was performed at the Department of Neurology, University Hospital of Split. Functional disability assessments of PPMS subjects were evaluated by an experienced neurologist who applied the EDSS. PB analyses were conducted at the Department of Medical Chemistry and Biochemistry, University of Split, School of Medicine. 

### 2.3. Flow Cytometry

Blood samples for flow cytometry analysis were collected from the antecubital veins of PPMS subjects after they signed an informed consent form. In the first test tube, one hundred microliters of whole blood were incubated for 20 min in a dark environment at 25 °C using the following mixture of antibodies: 4 µL of phycoerythrin-conjugated antibodies targeting human CD16 (BD Pharmingen, San Diego, CA, USA); 4 µL of FITC-conjugated antihuman-CD14 antibodies (from BD Pharmingen, San Diego, CA, USA); 3 µL of BB700-conjugated mouse antibodies specific to human CD192 (provided by BD Horizon, San Diego, CA, USA); and 5 µL of Alexa Fluor 647-conjugated antibodies against human CD40 (sourced from BD Pharmingen). In the second test tube, one hundred microliters of whole blood were incubated with 20 µL of phycoerythrin-conjugated antibodies reactive to human CD152 (CTLA-4) (BD Pharmingen, San Diego, CA, USA); 20 µL of antihuman-CD25 FITC antibodies (BD Pharmingen, San Diego, CA, USA); 5 µL of mouse antibodies reactive to human FOXP3 conjugated with BB700 (BD Horizon, San Diego, CA, USA); and 5 µL of Alexa Fluor 647 antibodies reactive to human CD4 (BD Pharmingen, San Diego, CA, USA). Following red blood cell lysis with BD Pharm Lyse^TM^ solution (BD Biosciences, San Diego, CA, USA), flow cytometric analyses were performed using a BD Accuri C6 (BD Biosciences, Aalst, Belgium). Unstained cell samples, together with samples stained with only one antibody, were measured and processed as negative controls to set the appropriate regions. Cell acquisition was halted at 10^6^ cells. The flow cytometry data for each marker was collected in one flow run.

Data acquired by cytometer were analysed using FlowLogic Software version 8 (Inivai Technologies, Melbourne, Australia). Monocytes (from the first test tube) and lymphocytes (from the second test tube) were recognized in the forward scatter/side scatter (FSC/SSC) dot plots. The FSC parameter indicates cell diameter, while SSC indicates cell granularity.

### 2.4. Statistical Analysis

For continuous parametric variables, the data were expressed as mean ± standard deviation. For continuous nonparametric variables, the median (interquartile range) was used. Categorical variables were presented as whole numbers and percentages. Student’s t-test was applied to analyze differences in continuous parametric variables, while the chi-squared test was used to compare categorical variables across different groups. Groups were compared by one-way ANOVA. Correlation analyses were conducted using the Spearman rank-order correlation coefficient (ρ).

All statistical analyses were performed using Past software (version 3.14, University of Oslo, Oslo, Norway) with the significance set at *p* < 0.05.

## 3. Results

### 3.1. Flow Cytometry Results on Lymphocytes of PPMS and HC Subjects

**Figure 1:**
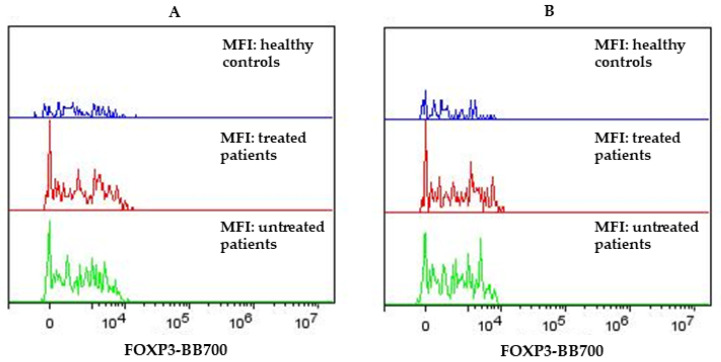
MFI of FOXP3 on CD4^+^CD25^+^^high^ (**A**) and CD4^+^CD25^+^ (**B**) in HCs and PPMS subjects (*p* < 0.05; *p* < 0.01).

All PPMS subjects’ findings differed from the HCs in the percentage of CD4^+^CD25^+high^, CD4^+^CD25^+^, and surface expression of CD4^+^CD25^+^^high^FOXP3^+^ and CD4^+^CD25^+^FOXP3^+^ (Table 2). Figure 1 presents median fluorescence intensity (MFI) increased surface expression of CD4^+^CD25^+^^high^FOXP3^+^ (A) and CD4^+^CD25^+^FOXP3^+^ (B) in PPMS subjects (treated and untreated), compared to the HCs. Untreated PPMS subjects had significantly increased percentages of CD4^+^CD25^+^^high^ (*p* < 0.01), as well as significantly increased percentages of CD4^+^CD25^+^ (*p* = 0.05). All PPMS subjects’ findings differed from the HCs in their percentages of CD4^+^CD25^+^^high^FOXP3^+^ and CD4^+^CD25^+^FOXP3^+^. Untreated PPMS subjects had significantly increased percentages of CD4^+^CD25^+^^high^FOXP3^+^ (*p* < 0.05), as well as significantly increased percentages of CD4^+^CD25^+^FOXP3^+^ (*p* < 0.001). Treated PPMS subjects had significantly increased percentages of CD4^+^CD25^+^FOXP3^+^ (*p* < 0.01) but had no significantly increased percentages of CD4^+^CD25^+high^FOXP3^+^ when compared to HCs (Table 2).

Treated PPMS subjects had significantly increased surface expression of CTLA-4^+^ in CD4^+^CD25^-^ (*p* < 0.05) compared to HCs (Table 3). Untreated PPMS subjects had significantly increased percentages of CD4^+^CD25^-^CTLA-4^+^ (*p* < 0.05) compared to HCs. All PPMS subjects’ findings differed from the HCs in the percentages of CD4^+^CD25^+^_,_ FOXP3^+^, and CTLA-4^+^ (*p* < 0.005) (Table 3). Treated PPMS subjects had significantly increased percentages of CD4^+^CD25^+^, FOXP3^+^, and CTLA-4^+^ (*p* < 0.004) compared with untreated PPMS subjects (*p* < 0.01) (Table 3). 

### 3.2. Flow Cytometry Results for Monocytes of PPMS and HC Subjects

All PPMS subjects’ findings differed from the HCs in the surface expression of CD192 in classical CD14^++^CD16^−^ monocytes, the percentages of CD14^++^CD16^+^ monocytes, and the percentages of CD40^+^ and CD192^+^ in nonclassical CD14^+^CD16^++^ monocytes differed between treated and untreated PPMS subjects (Table 4). All PPMS, treated and untreated, had significantly increased surface expression of CD192 in classical monocytes (both *p* < 0.001). 

**Figure 2:**
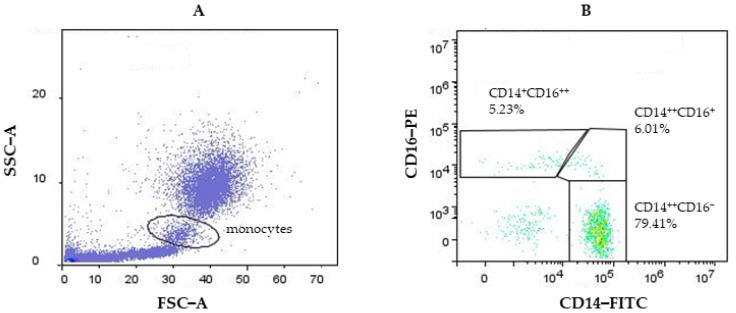
Representative gates for monocyte (**A**) and the monocyte subpopulations nonclassical (CD14^+^CD16^++^), intermediate (CD14^++^CD16^+^), and classical (CD14^++^CD16^−^) (**B**).

Monocyte subpopulations are shown in Figure 2. The cell population expressions of CD14 and CD16 were displayed in a plot to identify CD14^++^CD16^−^, CD14^++^CD16^+^, and CD14^+^CD16^++^ monocyte subsets. The gated subpopulation was analyzed for its percentage and surface receptor expression of CD40 and CD192. 

**Figure 3:**
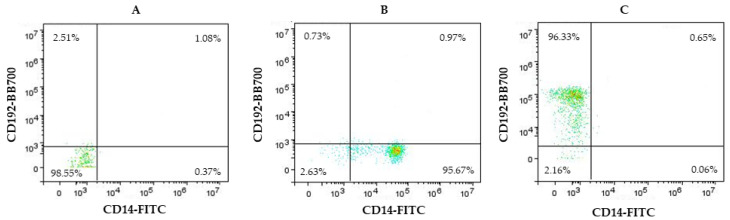
Representative dot plots for unstained sample (**A**), control total monocyte dot plots stained for CD14 (**B**), and only CD192 (**C**).

Figure 3 shows total monocyte control plots to monitor instrument setup and analysis strategy. 

**Figure 4:**
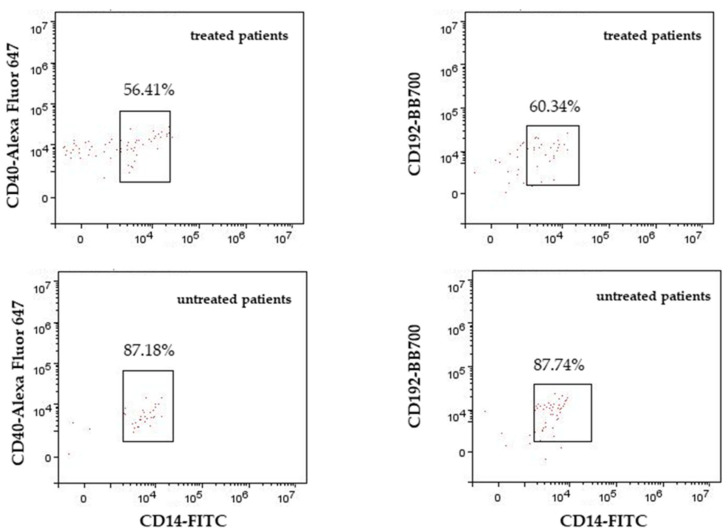
Percentages of CD40^+^ and CD192^+^ nonclassical monocytes in treated and untreated subjects. *p* < 0.01; *p* < 0.05.

Further, treated PPMS subjects in comparison with untreated PPMS subjects also had significantly decreased percentages of CD192 and CD40 in nonclassical monocytes (*p* < 0.05; and *p* < 0.01) (Figure 4).

## 4. Discussion

This study investigated the expression of CTLA-4 and FOXP3 in CD4^+^CD25^+^ Treg cells, and CD40 and CD192 in monocyte subpopulations in PPMS (ocrelizumab treated and untreated) subjects compared to healthy controls. The results showed significantly increased percentages of CTLA-4 and FOXP3 in CD4^+^CD25^+^ lymphocytes for PPMS subjects, both treated and untreated, significantly increased percentages of CD4^+^CD25^+high^FOXP3^+^ in untreated PPMS subjects and CD4^+^CD25^+^FOXP3^+^ in all PPMS subjects, and significantly decreased percentages of CD192^+^ and CD40^+^ nonclassical monocytes in treated PPMS subjects.

### 4.1. Discussion Related to Lymphocytes Results in PPMS and HC Subjects 

Tregs are CD4^+^ regulatory cells, which were discovered to be a unique population that inhibits the function of inflammatory cells. CD4^+^ Treg cells express several surface markers, including CD25, CD127 (negative-low), and FOXP3 [39]. CD25 is an IL-2 receptor α chain and is expressed abundantly on Tregs [30]. FOXP3, recognized as a master transcriptional regulator, is integral to the function and identity of regulatory T cells (Tregs). Its persistent expression is crucial for the maintenance of the suppressive functionality of mature, differentiated Treg cells. The expression of FOXP3 ensures that Tregs can effectively modulate immune responses, preventing autoimmune diseases and maintaining immune system homeostasis [40]. The mutation of FOXP3 in Tregs can induce a shift towards an autoimmune disease in both mice and humans [41,42]. The expression and mutation of FOXP3 highlight its pivotal role in maintaining the regulatory and suppressive characteristics of Tregs. Its absence can lead to the loss of these properties, resulting in the adoption of proinflammatory functions by these cells. 

In the present study, all PPMS subjects, treated and untreated, showed an increase in FOXP3 expression on CD4^+^CD25^high^ and also CD4^+^CD25^+^ Tregs compared with HCs, without significant differences between treated and untreated PPMS subjects. Treated PPMS subjects had slightly higher expression of FOXP3 on CD4^+^CD25^+^ compared to untreated ones. Li, Y.F. et al. [43] have performed a meta-analysis which included 16 studies, five of which identified Tregs as CD4+, CD 25+, and FOXP3+ cells. The studies in the review dated from the years 2009 to 2013. Pooling the data from these five studies showed that the proportion of Tregs in the MS patients appeared to be lower than in the controls. This meta-analysis had several limitations, such as the inclusion of various clinical subtypes of MS patients and different treatments of MS patients across the studies. These treatments may have had an influence on the proportion of Tregs in MS patients, and it is difficult to remove that influence from the results. The authors also stated that further studies are needed with independent cohorts of patients and larger sample sizes to validate their results, and that Tregs should be defined as CD4-positive, CD25-positive, and FOXP3-positive. The results from the present study also show differences in the percentages of Tregs. All PPMS subjects had significantly increased percentages of CD4^+^CD25^+^FOXP3^+^, while only untreated PPMS subjects had significantly increased percentages of CD4^+^CD25^high+^FOXP3^+^. Gonzales-Oria et al. [44] also found that percentages of Tregs (CD4^+^CD25^high^FOXP3^+^) were significantly higher in MS subjects (those with RRMS, PPMS, and CIS-clinical isolated syndrome). On the other hand, Kouchaki et al. [45] found a significantly lower frequency of CD4^+^CD25^+^FOXP3^+^ Tregs in MS subjects (with RRMS, PPMS, SPMS, CIS, and PRMS-progressive relapsing multiple sclerosis,) than in HCs, with the frequency of Tregs significantly higher in severe forms of MS (PPMS, SPMS, and PRMS) compared to the mild forms (CIS and RRMS) [44]. Ocrelizumab did not increase the percentage of CD4 Tregs over time, but markedly elevated the percentages of CD8 Tregs [46]. Chi et al. [20] documented the dysfunction of Tregs CD4^+^CD25^high^CD127^low^FOXP3^+^ cells, which play a key role in maintaining self-tolerance. 

Cytotoxic T lymphocyte-associated antigen 4 (CTLA-4) is a surface molecule of activated T cells that maintains the homeostasis of the immune system. It modulates immune responses by competitively binding to CD80 and CD86, obstructing CD28 interaction, which raises T cell activation thresholds, markedly diminishing immune activity [47]. CTLA-4 is expressed in Tregs but can also be upregulated in other T cell subsets, notably CD4^+^ T cells, following activation [48]. Exhausted T cells often express CTLA-4, among various inhibitory receptors. Exhausted T-cell responses are observed in various infections, including hepatitis B and C viruses, adenovirus, lymphocytic choriomeningitis virus, leukemia virus, polyoma virus, and Friend leukemia virus. Exhausted T-cell phenomenon is also noted in patients with malignancies [49]. In our study, all PPMS subjects showed an increased percentage of CD4^+^CD25^+^ FOXP3^+^ (Treg) CTLA-4^+^ compared to HCs. Treated PPMS subjects had more significant increases in the percentages of CD4^+^CD25^+^ FOXP3^+^ (Treg) CTLA-4^+^, compared to untreated PPMS subjects. There were no significant differences in the surface expression of CTLA-4 in CD4^+^CD25^+^FOXP3^+^ between all PPMS groups and HCs. However, our results differ from those of Sellebjerg et al. [50], who found lower percentages of CD4^+^CD25^high^ cells that were CTLA-4 positive in untreated RRMS patients and increased percentages of the same cells after IFN- β treatment. There was no correlation with the EDSS for lymphocytes and monocyte populations in PPMS subjects (treated and untreated) in the present study. Nevertheless, our finding of a more significant increase in the percentage of Treg CTLA-4^+^ cells in ocrelizumab treated PPMS subjects compared to untreated PPMS subjects corresponds to a slight EDSS decrease (6%) in ocrelizumab treated PPMS subjects. This suggests that CTLA-4, expressed on Treg cells, engages in trogocytosis to remove CD80/CD86 molecules from antigen-presenting cells, subsequently increasing the availability of programmed death ligand 1(PD-L1) on these cells. This process effectively reduces the presence of CD80/CD86, while enhancing free PD-L1 on the surface of antigen-presenting cells [32]. PD-L1, a molecule that plays a key role in autoantigen tolerance, can contribute to the suppression of abnormal immune responses in multiple sclerosis [51].

Untreated PPMS subjects showed increased percentages of CD4^+^CD25^-^CTLA-4^+^ and treated PPMS subjects showed increased expression of CTLA-4 on CD4^+^CD25^-^ cells. This could indicate the activity of CD4^+^ activated cells due to an attempt to regulate autoimmune responses, because CTLA-4, regardless of the cell type it is expressed on, downregulates CD80 and/or CD86 on APCs by binding to and removing these molecules, thereby inhibiting T cell activation and differentiation [52]. It could also be due to the exhaustion of T cells due to chronic disease [49]. Previous studies have reported contradictory results regarding the expression of CTLA-4 in MS patients compared with HCs. While some have reported that the expression of CTLA-4 is decreased in MS patients compared with HCs [53,54,55], others have found no significant difference [56,57,58], while Kosmaczewska et al. [59] reported an increased median percentage of freshly isolated peripheral blood CD4^+^ CTLA-4^+^ T cells in MS patients. The reason for these discrepancies could be that these studies [53,54,55,56,57,58,59] were mostly performed on various different forms of MS patients (RRMS, SPMS, PPMS, CIS, etc.), while none focused solely on PPMS subjects. 

### 4.2. Discussion Related to Monocyte Results in PPMS and HC Subjects 

**Figure 5:**
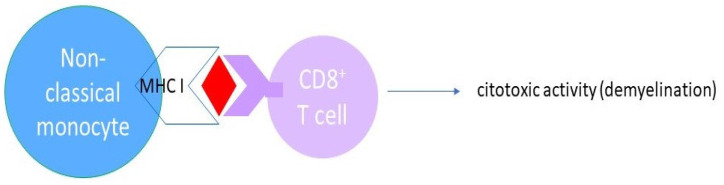
The role of nonclassical monocytes in mediating demyelination.

Monocytes are often divided into subpopulations (nonclassical CD14^+^CD16^++^, intermediate CD14^++^CD16^+^, and classical CD14^++^CD16^−^) depending on the expression of CD14 and CD16 [60]. Steinbach et al. [61] suggest that circulating monocytes and neutrophils produce inflammatory cytokines, leading to axonal damage [62]. It has been shown that the same cells, or certain subsets of classical monocytes and neutrophils, can actually oppose the initial activation and subsequent increase in pathogenic T cells [63]. CD16^+^ monocytes are described as being superior at activating T cells, suggesting that they are more active inducers of inflammation than the CD14^+^ monocytes [64] and that they can migrate through the blood–brain barrier more effectively than lymphocytes and CD14^+^ [65]. The findings of Waschbisch et al. [35] support the idea of the important role of CD16^+^ monocytes in shifting to sites of inflammation in the steady-state immune surveillance of the CNS, and they suggest that CD16^+^ monocytes cause the breakdown of the blood–brain barrier in CNS autoimmune diseases. Haschka et al. [66] reported that nonclassical monocyte proportions were elevated in RRMS subjects treated with natalizumab and suggested that myeloid cell immunophenotyping in MS may help to identify inactive RRMS earlier and facilitate monitoring of DMT response. Previous research in patients with systemic lupus and sepsis demonstrated that nonclassical monocytes have an inflammatory phenotype upon activation by high levels of proinflammatory cytokines and low levels of anti-inflammatory IL-10 [67]. The results from the present study show a significantly decreased percentage of CD40^+^, CD192^+^, and CD14^+^CD16^++^ monocytes for treated PPMS compared to untreated PPMS. Monocyte CD192 expression enables it to cross the blood–CNS barrier [68]. Further, monocyte CD40 binding to soluble CD40 ligands (CD40L) converts it to an antigen-presenting cell, which leads to T and B cell activation and CNS inflammation [69]. Therefore, our study finding of the decreased percentage of CD40^+^ and CD192^+^CD14^+^CD16^++^ monocytes probably indicates the beneficial effects of ocrelizumab therapy, which causes decreased entrance of monocytes into the CNS and decreased T and B cell activation compared to untreated PPMS subjects. In the present study, all PPMS subjects had decreased percentages of classical monocytes positive for CD192, but the surface expression of CD192 (MFI), both in untreated and treated PPMS subjects, was increased. The original studies of anti-CD20 antibodies in patients with multiple sclerosis assumed that depletion of CD20-expressing B cells may reduce elevated cerebrospinal fluid immunoglobulins [70]. Furthermore, small subsets of CD4^+^ and CD8^+^ T cells that also express CD20 can be depleted with anti-CD20 [71,72], indicating that anti-CD20 treatment directly removes pathogenic T CD20^+^. The presence of abnormally proinflammatory B cells in, which serve as antigen-presenting cells, in untreated MS patients was found to activate potentially pathogenic T cells and myeloid cells [73]. In addition, CD16^+^ nonclassical and intermediate monocytes can serve as antigen-presenting cells, which activate cytotoxic CD8^+^ T cells and destroy myelin [74]. Wong et al. [75] found the highest MHC class I (responsible for antigen presentation) expression in intermediate monocytes, and Zawada et al. [76] found their highest expression in nonclassical monocytes. Knowing these pathogenesis steps, we can assume the lower percentages of CD40^+^ and CD192^+^ nonclassical monocytes in treated patients in our study to be novel markers of the beneficial effect of anti-CD20 therapy (Figure 5).

The CD16^+^ monocyte subpopulations preferentially become migratory dendritic cells [77]. These CD16^+^ monocyte-derived cells may promote their survival as well as the survival and differentiation of CD16^-^ cells derived from classical monocytes. This means that some nonclassical monocyte subpopulations are superior and direct the destiny of major classical monocyte subpopulations. Recently, it was shown that the removal of T and B cells by anti-CD20 therapy alters their interactions in vivo [78]. Considering that CD40^+^ and CD192^+^ nonclassical monocytes lack the CD20 antigen, their decreased percentages in ocrelizumab-treated patients are the indirect results of altered T and B cell interactions.

Tregs express several surface markers and their persistent expression is crucial for maintaining the suppressive functionality of differentiated Treg cells (FOXP3) and effective modulation of the immune responses of CTLA-4. Their increased percentages can possibly contribute to inhibiting the abnormal immune response in PPMS. All PPMS subjects had significant increases in their percentages of CD4^+^CD25^+^FOXP3^+^, with greater significance levels in untreated PPMS subjects. Untreated PPMS subjects had a significant difference in CD4^+^CD25^+high^FOXP3^+^ compared to HCs, but treated PPMS subjects did not. This may prove that ocrelizumab suppresses autoimmune response, which is reflected in lower numbers of Tregs in ocrelizumab-treated PPMS subjects. Knowing that monocyte CD40 leads to T and B cell activation and CNS inflammation, and that monocyte CD192 enables monocytes to cross the blood–CNS barrier, decreased percentages of CD14^+^CD16^++^ monocytes may indicate a beneficial effect of ocrelizumab therapy. Lastly, our study has several limitations to mention. First, it comprises a relatively small number of PPMS subjects who could be enrolled in the study from the University Hospital of Split. Second, the possible effects of differences in MS disease duration between ocrelizumab treated and untreated PPMS subjects were not investigated due to the relatively small sample size. 

It is recommended that further studies include PPMS subjects with higher EDSS scores, control for the duration of the MS disease between ocrelizumab-treated and untreated PPMS subjects, and balance more appropriately the age of the healthy control subjects. Longitudinal follow-up is also recommended to gain more insights into the clinical relevance of the expression of CD40 and CD192 in monocytes and Treg lymphocytes in PPMS subjects.

## Figures and Tables

**Table 1:** Demographic and disease related characteristics of ocrelizumab treated and untreated PPMS subjects and HCs.

Parameter	All PPMS (N = 28)	Treated PPMS (N = 15)	Untreated PPMS (N = 13)	HCs (N = 10)
Mean ± SD				
Age (years)	54.57 ± 8.71	53 ± 7.47	56.3 ± 9.97	37 ± 13.90
EDSS	4.94 ± 1.54	4.8 ± 1.53	5.11 ± 1.6	/
Disease duration (years)	10.74 ± 7.58	6.86 ± 5.39	15.58 ± 7.26	/
Female/Male (N)	21/7	13/2	8/5	6/4

Basic parametric data are presented as mean ± standard deviation. Categorical data are presented as numbers. Abbreviations: PPMS—primary progressive MS; HCs—healthy controls; N—number of subjects.

**Table 2:** Lymphocyte marker expression in PPMS subjects and HCs.

		% of CD4^+^ CD25^+high^	MFI of CD4^+^ CD25^+high^	% of CD4^+^ CD25^+high^ FOXP3^+^	MFI of CD4^+^ CD25^+high^ FOXP3^+^	% of CD4^+^ CD25^+^	MFI of CD4^+^CD25^+^	% of CD4^+ ^ CD25^+^ FOXP3^+^	MFI of CD4^+^ CD25^+^ FOXP3^+^
Treated PPMS (N = 15)	M	3.02	3720.74	10.57	1798.26	5.502	36,098.34	21.89	1866.49
	SD	0.66	399.85	3.06	433.07	1.28	3422.33	5.41	474.53
HCs (N = 10)	M	2.56	3550.99	8.63	1417.0.3	4.54	34,907.46	15.48	1415.77
	SD	0.65	154.51	1.28	233.17	0.91	2195.62	2.63	209.94
Untreated PPMS (N = 13)	M	3.7	3679.51	11.06	1687.15	6.26	35,148.44	21.03	1744.54
	SD	1.08	215.02	2.68	258.27	1.76	3060.79	3.91	232.4
All PPMS (N = 28)	M	3.33	3701.6	10.76	1746.68	5.85	35,657.31	21.49	1809.87
	SD	0.93	322.32	2.89	360.6711	1.54	3235.67	4.71	380.26
All PPMS vs. HCs	t	2.4	1.14	2.22	2.69	2.47	0.67	3.8	3
	df	37	37	37	37	37	37	37	37
	*p*	0.02 *	0.16	0.03 *	0.01 *	0.01 *	0.05	0.0005 ***	0.003 **
Treated PPMS vs. HCs	t	1.7	1.27	1.88	2.55	1.99	0.97	3.46	2.81
	df	24	24	24	24	24	24	24	24
	*p*	0.1	0.21	0.07	0.01 *	0.058	0.34	0.002 **	0.009 **
Untreated PPMS vs. HCs	t	2.9	1.56	2.62	2.63	2.76	0.21	3.84	3.5
	df	22	22	22	22	22	22	22	22
	*p*	0.008 **	0.12	0.01*	0.01 *	0.01 *	0.83	0.0009 ***	0.002 **
Treated PPMS vs. Untreated PPMS	t	2	0.33	0.43	0.8	1.32	0.76	0.47	0.84
	df	27	27	27	27	27	27	27	27
	*p*	0.053	0.74	0.66	0.42	0.19	0.44	0.63	0.4

Abbreviations: PPMS—primary progressive multiple sclerosis; HCs—healthy controls; %—percentage; MFI—median fluorescence intensity; M—arithmetic mean; SD—standard deviation; df—degree of freedom; t—*t*-test. * *p* < 0.05; ** *p* < 0.01; *** *p* < 0.001.

**Table 3:** CTLA-4 marker expression in CD4+CD25- lymphocytes and Tregs in PPMS subjects and HCs.

		% of CD4^+^CD25^−^CTLA-4^+^	MFI of CTLA-4^+^ in CD4^+^CD25^−^	% of CD4^+^ CTLA-4^+^	MFI of CD4^+^ CTLA-4^+^	% of CD4^+^CD25^+^ FOXP3^+^ CTLA-4^+^	MFI of CD4^+^CD25^+^ FOXP3^+^ CTLA-4^+^
Treated PPMS (N = 15)	M	39.96	1327.11	15.29	2742.4	34.01	2378.26
	SD	9.59	196.92	4.34	144.93	7.84	294.41
HCs (N = 10)	M	36.61	1179.32	12.65	2711.42	25.57	2291.86
	SD	7.01	60.62	3.51	194.87	3.89	294.41
Untreated PPMS (N = 13)	M	42.16	1278.63	14.86	2652.08	30.41	2372.1
	SD	5.67	151.23	3.72	124.01	4.99	259
All PPMS (N = 28)	M	40.98	1308.14	15.09	2700.47	32.34	2375.4
	SD	7.95	178.37	4.00	140.82	6.08	273.44
All PPMS vs. HCs	t	1.53	2.1	1.7	0.19	2.96	0.85
	df	37	31	37	37	37	36
	*p*	0.13	0.04 *	0.09	0.85	0.005 **	0.4
Treated PPMS vs. HCs	t	0.94	2.17	1.59	0.45	3.14	0.78
	df	24	22	24	22	24	23
	*p*	0.35	0.04 *	0.12	0.93	0.004 **	0.44
Untreated PPMS vs. HCs	t	2.09	1.82	1.44	0.89	2.53	0.79
	df	22	17	22	22	22	21
	*p*	0.04 *	0.08	0.16	0.38	0.01 *	0.43
Treated PPMS vs. Untreated PPMS	t	0.72	0.62	0.27	1.75	1.42	0.05
	df	27	22	27	27	27	27
	*p*	0.47	0.53	0.78	0.09	0.16	0.95

Abbreviations: PPMS—primary progressive multiple sclerosis; HCs—healthy controls; %—percentage; MFI—median fluorescence intensity; M—arithmetic mean; SD—standard deviation; df—degree of freedom; t—*t*-test. * *p* < 0.05; ** *p* < 0.01.

**Table 4:** The differences in monocyte marker expression in PPMS subjects and HC subjects.

		% of CD40^+^ CD14^++^CD16^−^	MFI of CD40 in CD14^++^CD16^−^	% of CD192^+^ CD14^++^CD16^−^	MFI of CD192^+^ in CD14^++^CD16^−^	% of CD14^++^ CD16^+^	% of CD40^+^ in CD14^++^CD16^+^	MFI of CD40^+^ in CD14^++^CD16^+^	% of CD40^+^ in CD14^+^CD16^++^	% of CD192^+^ in CD14^+^CD16^++^
Treated PPMS (N = 15)	M	60.48	4200.943	83.92	101,066.2	7.37	79.59	9922.6	61.09	62.82
	SD	23.26	1398.083	9.665	8739.41	4.59	16.34	3149.26	11.67	15.3
HC (N = 10)	M	49.82	3442.95	97.77	81,837.85	3.53	83.274	8774.08	65.4	70.3
	SD	19.96	796.503	1.53	8242.14	2.37	6.36	2163.78	20.32	20.95
Untreated PPMS (N = 13)	M	52.142	3596.253	84.1	95,454.87	6.11	83.02	10,381.84	75.69	76.19
	SD	19.20	751.187	13.04	10,285.6	4.6	18.16	4155.25	13.61	13.13
All PPMS (N = 28)	M	56.61	3920.19	84.003	98,461	6.79	81.18	10,135.82	67.58	68.76
	SD	21.5	1165.84	11.13	9733.66	6.75	16.97	3586.68	14.36	15.65
All PPMS vs. HCs	t	0.87	1.19	3.9	4.8	2.11	0.37	1.12	0.36	0.24
	df	37	37	37	37	37	37	37	36	36
	*p*	0.38	0.24	0.0004 **	0.00002 ***	0.041 *	0.7	0.26	0.7	0.81
Treated PPMS vs. HCs	t	1.19	1.5	4.46	5.5	2.42	0.67	1	0.67	1.03
	df	24	24	24	24	24	24	24	24	24
	*p*	0.24	0.13	0.0001 ***	0.00001 ***	0.02 *	0.5	0.32	0.5	0.31
Untreated PPMS vs. HCs	t	0.28	0.47	3.28	3.4	−1.56	0.04	1.1	1.41	0.8
	df	22	22	22	22	22	22	22	21	21
	*p*	0.77	0.64	0.003*	0.002**	0.13	0.96	0.27	0.17	0.43
Treated PPMS vs. Untreated PPMS	t	1.02	1.3	0.04	1.5	0.71	0.52	0.33	3	2.39
	df	27	27	27	27	27	27	27	36	26
	*p*	0.31	0.17	0.96	0.13	0.48	0.6	0.74	0.006 ***	0.02 *

Abbreviations: PPMS—primary progressive multiple sclerosis; HCs—healthy controls; %—percentage; MFI—median fluorescence intensity; M—arithmetic mean; SD—standard deviation; df—degree of freedom; t—*t*-test. * *p* < 0.05; ** *p* < 0.01; *** *p* < 0.001.

## Data Availability

Further information regarding the resources and data availability should be directed to the corresponding author.

## References

[1] Axisa P.P., Hafler D.A. (2016). Multiple sclerosis: Genetics, biomarkers, treatments. Curr. Opin. Neurol..

[2] Hafler D.A. (2004). Multiple sclerosis. J. Clin. Investig..

[3] Klineova S., Lublin F.D. (2018). Clinical Course of Multiple Sclerosis. Cold Spring Harb. Perspect. Med..

[4] McGinley M.P., Goldschmidt C.H., Rae-Grant A.D. (2021). Diagnosis and Treatment of Multiple Sclerosis: A Review. JAMA.

[5] Lassmann H., Brück W., Lucchinetti C. (2001). Heterogeneity of multiple sclerosis pathogenesis: Implications for diagnosis and therapy. Trends Mol. Med..

[6] Pitteri M., Romualdi C., Magliozzi R., Monaco S., Calabrese M. (2017). Cognitive impairment predicts disability progression and cortical thinning in MS: An 8-year study. Mult. Scler..

[7] Goverman J. (2009). Autoimmune T cell responses in the central nervous system. Nat. Rev. Immunol..

[8] Dendrou C.A., Fugger L., Friese M.A. (2015). Immunopathology of multiple sclerosis. Nat. Rev. Immunol..

[9] van Langelaar J., Rijvers L., Smolders J., van Luijn M.M. (2020). B and T Cells Driving Multiple Sclerosis: Identity, Mechanisms and Potential Triggers. Front. Immunol..

[10] Monti P., Heninger A.K., Bonifacio E. (2009). Differentiation, expansion, and homeostasis of autoreactive T cells in type 1 diabetes mellitus. Curr. Diabetes Rep..

[11] Iijima W., Ohtani H., Nakayama T., Sugawara Y., Sato E., Nagura H., Yoshie O., Sasano T. (2003). Infiltrating CD8+ T cells in oral lichen planus predominantly express CCR5 and CXCR3 and carry respective chemokine ligands RANTES/CCL5 and IP-10/CXCL10 in their cytolytic granules: A potential self-recruiting mechanism. Am. J. Pathol..

[12] Cao Y., Amezquita R.A., Kleinstein S.H., Stathopoulos P., Nowak R.J., O’Connor K.C. (2016). Autoreactive T Cells from Patients with Myasthenia Gravis Are Characterized by Elevated IL-17, IFN-γ, and GM-CSF and Diminished IL-10 Production. J. Immunol..

[13] Diani M., Altomare G., Reali E. (2015). T cell responses in psoriasis and psoriatic arthritis. Autoimmun. Rev..

[14] Schreurs O., Karatsaidis A., Schenck K. (2016). Phenotypically non-suppressive cells predominate among FOXP3-positive cells in oral lichen planus. J. Oral. Pathol. Med..

[15] Xufré C., Costa M., Roura-Mir C., Codina-Busqueta E., Usero L., Pizarro E., Obiols G., Jaraquemada D., Martí M. (2013). Low frequency of GITR+ T cells in ex vivo and in vitro expanded Treg cells from type 1 diabetic patients. Int. Immunol..

[16] Richetta A.G., Mattozzi C., Salvi M., Giancristoforo S., D’epiro S., Milana B., Carboni V., Zampetti M., Calvieri S., Morrone S. (2011). CD4+CD25+ T-regulatory cells in psoriasis. Correlation between their numbers and biologics-induced clinical improvement. Eur. J. Dermatol..

[17] Su Q.Y., Zhang S.X., Yang L., Luo J., Li X.F., Zhang J.Q., Zhang Y., Liu J.Q., Shi L. (2023). Peripheral Treg Levels and Transforming Growth Factor-β (TGFβ) in Patients with Psoriatic Arthritis: A Systematic Review Meta-Analysis. Adv. Ther..

[18] Thiruppathi M., Rowin J., Li Jiang Q., Sheng J.R., Prabhakar B.S., Meriggioli M.N. (2012). Functional defect in regulatory T cells in myasthenia gravis. Ann. N. Y. Acad. Sci..

[19] Kohler S., Keil T.O.P., Hoffmann S., Swierzy M., Ismail M., Rückert J.C., Alexander T., Meisel A. (2017). CD4+ FOXP3+ T regulatory cell subsets in myasthenia gravis patients. Clin. Immunol..

[20] Chi L.J., Wang H.B., Zhang Y., Wang W.Z. (2007). Abnormality of circulating CD4^+^ CD25^+^ regulatory T cell in patients with Guillain-Barré syndrome. J. Neuroimmunol..

[21] Tapia-Maltos M.A., Treviño-Frenk I., García-González H.B., Rosetti M., Barriga-Maldonado V., Morales-Ramírez F., López-Hernández D.C., Rosetti F., Crispín J.C. (2021). Identification of regulatory T cell molecules associated with severity of multiple sclerosis. Mult. Scler..

[22] Dalla Libera D., Di Mitri D., Bergami A., Centonze D., Gasperini C., Grasso M.G., Galgani S., Martinelli V., Comi G., Avolio C. (2011). T regulatory cells are markers of disease activity in multiple sclerosis patients. PLoS ONE.

[23] Bjerg L., Brosbøl-Ravnborg A., Tørring C., Dige A., Bundgaard B., Petersen T., Höllsberg P. (2012). Altered frequency of T regulatory cells is associated with disability status in relapsing-remitting multiple sclerosis patients. J. Neuroimmunol..

[24] Viglietta V., Baecher-Allan C., Weiner H.L., Hafler D.A. (2004). Loss of functional suppression by CD4+CD25+ regulatory T cells in patients with multiple sclerosis. J. Exp. Med..

[25] Venken K., Hellings N., Thewissen M., Somers V., Hensen K., Rummens J.L., Medaer R., Hupperts R., Stinissen P. (2008). Compromised CD4+ CD25(high) regulatory T-cell function in patients with relapsing-remitting multiple sclerosis is correlated with a reduced frequency of FOXP3-positive cells and reduced FOXP3 expression at the single-cell level. Immunology.

[26] Haas J., Hug A., Viehöver A., Fritzsching B., Falk C.S., Filser A., Vetter T., Milkova L., Korporal M., Fritz B. (2005). Reduced suppressive effect of CD4+CD25high regulatory T cells on the T cell immune response against myelin oligodendrocyte glycoprotein in patients with multiple sclerosis. Eur. J. Immunol..

[27] Li R., Li H., Yang X., Hu H., Liu P., Liu H. (2022). Crosstalk between dendritic cells and regulatory T cells: Protective effect and therapeutic potential in multiple sclerosis. Front. Immunol..

[28] Colamatteo A., Carbone F., Bruzzaniti S., Galgani M., Fusco C., Maniscalco G.T., Di Rella F., de Candia P., De Rosa V. (2020). Molecular Mechanisms Controlling FOXp3 Expression in Health and Autoimmunity: From Epigenetic to Post-translational Regulation. Front. Immunol..

[29] Eggenhuizen P.J., Ng B.H., Ooi J.D. (2020). Treg Enhancing Therapies to Treat Autoimmune Diseases. Int. J. Mol. Sci..

[30] Nie J., Li Y.Y., Zheng S.G., Tsun A., Li B. (2015). FOXP3(+) Treg Cells and Gender Bias in Autoimmune Diseases. Front. Immunol..

[31] Danikowski K.M., Jayaraman S., Prabhakar B.S. (2017). Regulatory T cells in multiple sclerosis and myasthenia gravis. J. Neuroinflamm..

[32] Tekguc M., Wing J.B., Osaki M., Long J., Sakaguchi S. (2021). Treg-expressed CTLA-4 depletes CD80/CD86 by trogocytosis, releasing free PD-L1 on antigen-presenting cells. Proc. Natl. Acad. Sci. USA.

[33] Calahorra L., Camacho-Toledano C., Serrano-Regal M.P., Ortega M.C., Clemente D. (2022). Regulatory Cells in Multiple Sclerosis: From Blood to Brain. Biomedicines.

[34] Carstensen M., Christensen T., Stilund M., Møller H.J., Petersen E.L., Petersen T. (2020). Activated monocytes and markers of inflammation in newly diagnosed multiple sclerosis. Immunol. Cell Biol..

[35] Waschbisch A., Schröder S., Schraudner D., Sammet L., Weksler B., Melms A., Pfeifenbring S., Stadelmann C., Schwab S., Linker R.A. (2016). Pivotal Role for CD16+ Monocytes in Immune Surveillance of the Central Nervous System. J. Immunol..

[36] Gjelstrup M.C., Stilund M., Petersen T., Møller H.J., Petersen E.L., Christensen T. (2018). Subsets of activated monocytes and markers of inflammation in incipient and progressed multiple sclerosis. Immunol. Cell Biol..

[37] Dargahi N., Katsara M., Tselios T., Androutsou M.E., de Courten M., Matsoukas J., Apostolopoulos V. (2017). Multiple Sclerosis: Immunopathology and Treatment Update. Brain Sci..

[38] Hauser S.L., Cree B.A.C. (2020). Treatment of Multiple Sclerosis: A Review. Am. J. Med..

[39] Kimura K. (2020). Regulatory T cells in multiple sclerosis. Clin. Exp. Neuroimmunol..

[40] Williams L.M., Rudensky A.Y. (2007). Maintenance of the FOXp3-dependent developmental program in mature regulatory T cells requires continued expression of FOXp3. Nat. Immunol..

[41] Ben-Skowronek I. (2021). IPEX Syndrome: Genetics and Treatment Options. Genes.

[42] Leon J., Chowdhary K., Zhang W., Ramirez R.N., André I., Hur S., Mathis D., Benoist C. (2023). Mutations from patients with IPEX ported to mice reveal different patterns of FOXP3 and Treg dysfunction. Cell Rep..

[43] Li Y.F., Zhang S.X., Ma X.W., Xue Y.L., Gao C., Li X.Y., Xu A.D. (2019). The proportion of peripheral regulatory T cells in patients with Multiple Sclerosis: A meta-analysis. Mult. Scler. Relat. Disord..

[44] González-Oria M.C., Márquez-Coello M., Girón-Ortega J.A., Argente J., Moya M., Girón-González J.A. (2019). Monocyte and Lymphocyte Activation and Regulation in Multiple Sclerosis Patients. Therapy Effects. J. Neuroimmune Pharmacol. Off. J. Soc. Neuroimmune Pharmacol..

[45] Kouchaki E., Salehi M., Reza Sharif M., Nikoueinejad H., Akbari H. (2014). Numerical status of CD4^+^CD25^+^FOXP3^+^ and CD8^+^CD28^−^ regulatory T cells in multiple sclerosis. Iran. J. Basic. Med. Sci..

[46] Howlett-Prieto Q., Feng X., Kramer J.F., Kramer K.J., Houston T.W., Reder A.T. (2021). Anti-CD20 therapy corrects a CD8 regulatory T cell deficit in multiple sclerosis. Mult. Scler..

[47] Seidel J.A., Otsuka A., Kabashima K. (2018). Anti-PD-1 and Anti-CTLA-4 Therapies in Cancer: Mechanisms of Action, Efficacy, and Limitations. Front. Oncol..

[48] Chan D.V., Gibson H.M., Aufiero B.M., Wilson A.J., Hafner M.S., Mi Q.S., Wong H.K. (2014). Differential CTLA-4 expression in human CD4+ versus CD8+ T cells is associated with increased NFAT1 and inhibition of CD4+ proliferation. Genes Immun..

[49] Yi J.S., Cox M.A., Zajac A.J. (2010). T-cell exhaustion: Characteristics, causes and conversion. Immunology.

[50] Sellebjerg F., Krakauer M., Khademi M., Olsson T., Sørensen P.S. (2012). FOXP3, CBLB and ITCH gene expression and cytotoxic T lymphocyte antigen 4 expression on CD4^+^ CD25^high^ T cells in multiple sclerosis. Clin. Exp. Immunol..

[51] Medina S., Sainz de la Maza S., Villarrubia N., Álvarez-Lafuente R., Costa-Frossard L., Arroyo R., Monreal E., Tejeda-Velarde A., Rodríguez-Martín E., Roldán E. (2019). Teriflunomide induces a tolerogenic bias in blood immune cells of MS patients. Ann. Clin. Transl. Neurol..

[52] Rabe H., Lundell A.C., Andersson K., Adlerberth I., Wold A.E., Rudin A. (2011). Higher proportions of circulating FOXP3+ and CTLA-4+ regulatory T cells are associated with lower fractions of memory CD4+ T cells in infants. J. Leukoc. Biol..

[53] Mohammadzadeh A., Rad I.A., Ahmadi-Salmasi B. (2018). CTLA-4, PD-1 and TIM-3 expression predominantly downregulated in MS patients. J. Neuroimmunol..

[54] Wang H., Wang K., Zhong X., Dai Y., Wu A., Li Y., Hu X. (2012). Plasma sCD28, sCTLA-4 levels in neuromyelitis optica and multiple sclerosis during relapse. J. Neuroimmunol..

[55] Derakhshani A., Asadzadeh Z., Safarpour H., Leone P., Shadbad M.A., Heydari A., Baradaran B., Racanelli V. (2021). Regulation of CTLA-4 and PD-L1 Expression in Relapsing-Remitting Multiple Sclerosis Patients after Treatment with Fingolimod, IFNβ-1α, Glatiramer Acetate, and Dimethyl Fumarate Drugs. J. Pers. Med..

[56] Mena E., Rohowsky-Kochan C. (1999). Expression of costimulatory molecules on peripheral blood mononuclear cells in multiple sclerosis. Acta Neurol. Scand..

[57] Lavon I., Heli C., Brill L., Charbit H., Vaknin-Dembinsky A. (2019). Blood Levels of Co-inhibitory-Receptors: A Biomarker of Disease Prognosis in Multiple Sclerosis. Front. Immunol..

[58] Oliveira E.M., Bar-Or A., Waliszewska A.I., Cai G., Anderson D.E., Krieger J.I., Hafler D.A. (2003). CTLA-4 dysregulation in the activation of myelin basic protein reactive T cells may distinguish patients with multiple sclerosis from healthy controls. J. Autoimmun..

[59] Kosmaczewska A., Bilinska M., Ciszak L., Noga L., Pawlak E., Szteblich A., Podemski R., Frydecka I. (2007). Different patterns of activation markers expression and CD4+ T-cell responses to ex vivo stimulation in patients with clinically quiescent multiple sclerosis (MS). J. Neuroimmunol..

[60] Bar-Or A. (2008). The immunology of multiple sclerosis. Semin. Neurol..

[61] Steinbach K., Piedavent M., Bauer S., Neumann J.T., Friese M.A. (2013). Neutrophils amplify autoimmune central nervous system infiltrates by maturing local APCs. J. Immunol..

[62] Ajami B., Bennett J.L., Krieger C., McNagny K.M., Rossi F.M.V. (2011). Infiltrating monocytes trigger EAE progression, but do not contribute to the resident microglia pool. Nat. Neurosci..

[63] Mitsdoerffer M., Schreiner B., Kieseier B.C., Neuhaus O., Dichgans J., Hartung H.P., Weller M., Wiendl H. (2005). Monocyte-derived HLA-G acts as a strong inhibitor of autologous CD4 T cell activation and is upregulated by interferon-β in vitro and in vivo: Rationale for the therapy of multiple sclerosis. J. Neuroimunol..

[64] Ziegler-Heitbrock L. (2007). The CD14þ CD16þ blood monocytes: Their role in infection and inflammation. J. Leukoc. Biol..

[65] Chuluundorj D., Harding S.A., Abernethy D., La Flamme A.C. (2014). Expansion and preferential activation of the CD14^+^CD16^+^ monocyte subset during multiple sclerosis. Immunol. Cell Biol..

[66] Haschka D., Tymoszuk P., Bsteh G., Petzer V., Berek K., Theurl I., Berger T., Weiss G. (2020). Expansion of Neutrophils and Classical and Nonclassical Monocytes as a Hallmark in Relapsing-Remitting Multiple Sclerosis. Front. Immunol..

[67] Mukherjee R., Kanti Barman P., Kumar Thatoi P., Tripathy R., Kumar Das B., Ravindran B. (2015). Non-Classical monocytes display inflammatory features: Validation in Sepsis and Systemic Lupus Erythematous. Sci. Rep..

[68] Paré A., Mailhot B., Lévesque S.A., Juzwik C., Ignatius Arokia Doss P.M., Lécuyer M.A., Prat A., Rangachari M., Fournier A., Lacroix S. (2018). IL-1β enables CNS access to CCR2hi monocytes and the generation of pathogenic cells through GM-CSF released by CNS endothelial cells. Proc. Natl. Acad. Sci. USA.

[69] Chitnis T., Khoury S.J. (2003). Role of costimulatory pathways in the pathogenesis of multiple sclerosis and experimental autoimmune encephalomyelitis. J. Allergy Clin. Immunol..

[70] Breij E.C., Brink B.P., Veerhuis R., Van den Berg C., Vloet R., Yan R., Dijkstra C.D., Van der Valk P., Bö L. (2008). Homogeneity of active demyelinating lesions in established multiple sclerosis. Ann. Neurol..

[71] Von Essen M.R., Ammitzbøll C., Hansen R.H., Petersen E.R.S., McWilliam O., Marquart H.V., Damm P., Sellebjerg F. (2019). Proinflammatory CD20+ T cells in the pathogenesis of multiple sclerosis. Brain.

[72] Sabatino J.J., Wilson M.R., Calabresi P.A., Hauser S.L., Schneck J.P., Zamvil S.S. (2019). Anti-CD20 therapy depletes activated myelin-specific CD8^+^ T cells in multiple sclerosis. Proc. Natl. Acad. Sci. USA.

[73] Bar-Or A., Fawaz L., Fan B., Darlington P.J., Rieger A., Ghorayeb C., Calabresi P.A., Waubant E., Hauser S.L., Zhang J. (2010). Abnormal B-cell cytokine responses a trigger of T-cell-mediated disease in MS?. Ann. Neurol..

[74] Wong K.L., Yeap W.H., Tai J.J., Ong S.M., Dang T.M., Wong S.C. (2012). The three human monocyte subsets: Implications for health and disease. Immunol. Res..

[75] Wong K.L., Tai J.J., Wong W.C., Han H., Sem X., Yeap W.H., Kourilsky P., Wong S.C. (2011). Gene expression profiling reveals the defining features of the classical, intermediate, and nonclassical human monocyte subsets. Blood.

[76] Zawada A.M., Rogacev K.S., Rotter B., Winter P., Marell R.R., Fliser D., Heine G.H. (2011). SuperSAGE evidence for CD14++CD16+ monocytes as a third monocyte subset. Blood.

[77] Randolph G.J., Sanchez-Schmitz G., Liebman R.M., Schäkel K. (2002). The CD16^+^ (FcγRIII^+^) subset of human monocytes preferentially becomes migratory dendritic cells in a model tissue setting. J. Exp. Med..

[78] Shinoda K., Li R., Rezk A., Mexhitaj I., Patterson K.R., Kakara M., Zuroff L., Bennett J.L., von Büdingen H.C., Carruthers R. (2023). Differential effects of anti-CD20 therapy on CD4 and CD8 T cells and implication of CD20-expressing CD8 T cells in MS disease activity. Proc. Natl. Acad. Sci. USA.

